# ‘With my parents I can tell them anything’: intimacy levels within British Chinese families

**DOI:** 10.1080/02673843.2014.884007

**Published:** 2014-04-23

**Authors:** Carmen Lau Clayton

**Affiliations:** School of Sociology and Social Policy, University of Leeds, Leeds, UK

**Keywords:** British Chinese, parents, children, family, intimacy

## Abstract

Intimacy within relationships and the parent–child bond in particular is said to provide feelings of acceptance, warmth, sensitivity and an appreciation of self and can impact positively upon health and well-being of individuals. Views of intimacy can differ across cultures however, and may not be universally shared or understood. Accordingly, societies will have differing perspectives on how intimacy should be displayed between parents and children. For contemporary Chinese households living in Britain, we do not have an understanding of how residency in the UK may impact upon traditional Chinese values in terms of parent–child intimacy levels. Through a qualitative study with a set of 12 diverse Chinese families living in Britain, using repeat interviews over a nine-month period, Chinese parents and children were asked to reflect on past and present childhood experiences to uncover the ways in which intimacy is displayed and promoted within the parent–child dyad.

The pursuit of human relationships and connecting with others is a universal and common experience. Seen as one of the most important features of life, relationships have been described as the raw materials by and through which personal biography; family and social structure are framed and constructed (Cooney, 2000). When discussing intimacy within a relationship, it is generally associated with sex and sexuality. This can be attributed to early analytical work, where the focus was upon intimacy and sexual relating within heterosexual couplings, often within the context of marriage (Gabb, 2008). However, within a broader understanding, intimacy is frequently related to display of love, affection, trust and cohesiveness between friends, family and other close associates. If we view intimate relations as involving various interactions and familiarity between two people (such as the display of love, cohesion, attachment and connectedness), then intimacy is arguably found within most relationship types, including the parent–child attachment.

Parent–child relationships are portrayed as the most intimate and significant attachment due to profound psychological and social bonds. Within attachment theory, for example, parent–child relationships are postulated as being the starting point for experiencing intimacy and laying down the foundations for intimate understanding (Bowlby, 1969). Intimacy can provide feelings of acceptance, warmth, sensitivity and an appreciation of self (Prager, 1995) and impact positively upon health and well-being (Kawachi & Berkman, 2001). Furthermore, intimacy within a relationship can exert a direct and constructive influence upon the relationship functioning because of its own reward value (Reis & Shaver, 1988).

Views of intimacy can differ across cultures however, and may not be universally shared or understood (Broude, 1987). Accordingly, societies will have differing perspectives on how intimacy should be displayed between parents and children. Within Western or individualist societies, such as the UK, the construction of self as a separate and autonomous being coincides with the values placed upon the exploration and expression of individual feelings. As such, self-disclosure, physical and emotional expressiveness are ways in which parents and children can demonstrate intimacy towards one another. In contrast, collectivist societies, such as Chinese and Confucian culture, emphasise on the well-being and harmony of the group, such as the family, society and the state, over the individual. Within Chinese norms then, the fulfilment of an individual's needs for intimate relating within a Western framework may be viewed as self-indulgent and a source of shame (Prager, 1995). Inhibition and self-restraint are also considered as the indices of accomplishment, mastery and maturity within Confucian philosophy (King & Bond, 1985). As a result, Chinese parents are said to be less physically and emotionally expressive with their children. Instead, love is demonstrated by successfully meeting the child's needs (Uba, 1994). For contemporary Chinese households living in Britain, migrant or otherwise, we do not have an understanding of how residency in the UK may impact upon traditional Chinese values in terms of parent–child intimacy levels. Despite being the third largest and fastest growing UK ethnic minority group (Baxter & Raw, 2002; Office for National Statistics, 2012), research interest in and political focus on the British Chinese community do not reflect their long-standing presence. Through a qualitative study with a set of 12 diverse British Chinese families, using repeat interviews over a nine-month period, Chinese parents and children were asked to reflect on past and present childhood experiences to uncover the ways in which intimacy is displayed and promoted within the parent–child dyad. By collecting data from both family members’ perspectives, a deeper understanding of the complexities and nuances of modern British Chinese life can be revealed.

## Method, sample and analysis

The exploration of contemporary British Chinese family life was based on an Economic and Social Research Council funded doctoral study undertaken with 12 British Chinese households. The sample size was kept small to acquire an in-depth and rich account of Chinese individual's lives and experiences. By using a small sample, more meaningful relationships can be developed and insightful data can be collected (Silverman, 2011). Seven families were invited to take part in the study through Chinese organisations such as Chinese language supplementary schools and community groups. The other five families were snowballed from the initially recruited sample. All 12 Chinese families were from the North of England.

In each family, one parent and one child were interviewed separately either at home or within Chinese community centres. By using a one-to-one interviewing technique, the researcher was able to engage and adapt the interview process in accordance with the respondent's requirements and comfort. In 2008, each parent and child participated in three repeat interviews over a nine-month period, resulting in 72 interviews in total. Each interview was conducted two to three months apart and was based upon the study's three research interests: (1) parenting approaches, (2) parent–child intimacy levels and (3) children's agency. The interview scripts regarding family intimacy looked specifically at the presence and demonstration of closeness between the parent–child bonds. As intimacy can be conceived as a broad and overlapping concept, an imposed definition was not used in the research (as recommended by Berry, 1969). Instead, the research explored parent–child intimacy within a variety of behaviours, interactions and experiences which the British Chinese participants spoke of themselves.

Repeat qualitative interviewing was used as it allows participants to tell their stories on their own terms and in their own words. One-off interviews have been criticised for creating a limited ‘snapshot’ of data collection. Whereas, a series of interviews are better suited in capturing participant's thoughts and reflections (Ritchie & Lewis, 2003). Contact over time is also necessary in research for children and adults so that participants can relax enough with the researcher to reveal their beliefs, feelings and concerns (Hill, 2006). In turn, repeat interviews allow the interviewees to get to know the researcher better. As such, more of a reciprocal relationship can be formed between the researcher and respondent. The establishment of trust and reciprocity was hoped to elicit higher levels of disclosures from the interviewees and the production of authentic accounts. The success of qualitative research is highly dependent upon the formation and maintenance of personal relationships with the participants and establishing feelings of trust and rapport. Arguably, such a relationship would have been hard to create from a one-off interview. The process of returning to participants also allowed member checking of the results (Rizzo, Corsaro, & Bates, 1992). The use of three repetitive interviews with British Chinese families does not appear to have been used within previous research, where one-off interviews and questionnaire surveys have been predominantly used. Eight of the 12 families can be classified as a nuclear household. The sample also had one blended household, a female lone parent family and an ‘astronaut’ family (see Waters, 2005). Parents were from different countries of origin, including Mainland China, Hong Kong and Malaysia, and had varied lengths of UK residency (from those who were British born to a minimum of two years residency), education levels (primary education up to PhD doctorates) and careers (manual and professional employment). Nine mothers and three fathers between their late thirties and sixties participated in the study.

Children in the sample were aged between 11 and 14 years. Children of this age group were chosen due to their current and shared life experiences of the onset and early stages of adolescence and secondary schooling. Both adolescence and the movement to secondary school have been suggested to be a time of change and reflection for young people and their parents. Such factors were thought to have a possible impact or influence upon children's views of family relationships. Four boys and eight girls participated in the study. It was paramount that British Chinese children themselves were involved in expressing their views. This reflects the belief that children are important in generating knowledge and recognises the child's rights of expression (United Nations Convention on the Rights of the Child [UNCRC], 1989). Pseudonyms have been used to protect the identities of both children and parent within this article.

Parallel to the participation rights promulgated by the UNCRC (1989) are the writings from the New Sociology of Childhood, which views children as actors and agents (Greene & Hill, 2006). The New Sociology of Childhood starts from a broadly social constructionist premise and is related to the epistemological position of interpretivism. Interpretivism is an approach that emphasises the subjective meaning of social action. Accordingly, the interpretative position is concerned with drawing out the rich and unique experiences of individuals and how their social worlds are interpreted, understood and produced. Qualitative research is seen as complementary to the interpretative tradition, as methods are suggested to help describe and illuminate the participant's social world (Elwood & Martin, 2000).

The researcher personally transcribed the interview accounts, as it allowed for a deeper level of familiarity with the data-sets for the analysis process. Meaningful data from the transcripts were organised by themes and further subcategories, according to the research questions and links to pre-existing theories and research. The job of indexing, or slicing the data-set, was done manually with the aid of Microsoft Office. The combination of personally transcribing the interview accounts with the manual procedure of indexing allowed a more thorough examination of what the interviewees had said and permitted repeated examinations of their accounts. A conceptual map was subsequently developed on paper to interpret the data, where relevant interview verbatim was assigned to emerging themes. Analysis from the conceptual map then fed into the next stages of investigation for the following interview waves. The maintenance of a research diary was a method of logging research developments, research activities and general reflections. Such considerations also aided the design of subsequent interview guidelines. By documenting the data, the researcher's perspective and the decision-making process throughout the research, it allowed auditability and external reliability of the investigation (Shek, Tang, & Han, 2005). Together, the transcripts, thematic coding and conceptual map enabled a repetitive interplay between theory, data collection and analysis to be completed in an iterative manner, known as an abductive process. Within an abductive strategy, theory is constructed by first describing the activities and meanings by groups and individuals, followed by the formulation of categories and concepts to understand or explain the problem at hand (Blaikie, 2000). The emerging data categories and theories in this research were therefore seen as ‘thick descriptions’, where there was interpretive focus on the meaning and intentions of the interviewee's behaviours. The alternating process of moving between everyday concepts and meanings, lay accounts and social science explanations means that theory is therefore generated as an intimate part of the research process (Blaikie, 2000).

## Research findings: cultural differences

Within traditional and contemporary Chinese culture, achieving and maintaining social order and interpersonal harmony are key (Chen, Rubin, Li, & Li, 1999). Confucian doctrine stresses that one's good intentions are conveyed through actions more than words. As a result, Western understandings of warmth and care, which incorporate physical and emotional demonstration, may not be applicable to collectivist understandings that are based on support through involvement and investment (Lim & Lim, 2005). As one British Chinese father recalled in his childhood:

Whilst we were growing up, my parents priorities were making sure that we were all well fed, had enough, and had all the necessities needed to go through our childhood and the standard education system. (Ting, early forties, Chinese father)

Filial behaviours (e.g. being grateful and submissive to parents) are suggested to foster family harmony by lessening family conflict and increasing feelings of mutual obligation (Marshall, 2008). The child's adherence to authority is seen to encourage relatedness and responsiveness within the parent–child relationship (Rothbaum, Morelli, Pott, & Liu-Constant, 2000). In practice, however, expected filial behaviours can negatively affect intimate relating between Chinese parents and children, as seen within parents’ accounts of their upbringing.

My mother did love us, but it's not like now where you are physically affectionate towards your children. It just didn't happen. They just told you off regularly, but back then, telling off was to teach. (Sue, early fifties, Chinese mother)

Similarly, British Chinese children such as Olivia and Adam (both 12 years old) felt that their parents were ‘too traditional’ in their expectations and child-rearing methods. Children suggested that their parents’ expectation of filial piety was in contrast to Western norms and expectations. Parents were viewed as being unwilling to understand and accept the adolescent's needs with regards to personal freedoms, rights of expressions and intimate relating. As such, Chinese adolescents did not enjoy talking or opening up to their parents. Children also felt resentful that their feelings or values were not taken into account.

They never listen to me. I'm actually scared of my dad; he's so strict so I like to keep a distance. (Olivia, 12 years old)

Chinese parenting styles which are distant and formal stand in sharp contrast to the warmth and affection overtly expressed in individualistic societies. Consequently, British Chinese children may not recognise their parents’ love for them when they live in mainstream Western cultures (see Sung, 1985). In relation to cultural issues, Chinese language use within the home could also impact upon parent–child closeness. Four British Chinese children commented that language barriers resulted in less self-disclosure and reduced feelings of intimacy with parents.

I speak Chinese to my mum but then it's hard for me to speak Chinese to her, because obviously my English is better … My Chinese is OK but there are still some things I don't know how to say. I think if she was better at English we would be closer. (Sophie, 14 years old)

As a result of cultural differences and language difficulties, these children felt closer to other family relatives and friends. Often, children mentioned their relationships with younger and older siblings as being important and intimate. Sharing similar experiences with siblings, both within and outside of the home, was a contributory factor in the bonding experience.

I'd rather talk to my brother about things like school, friends and arguments about friends. I would tell my brother about it and ask him what to do? He's fallen out with his friends before as well, so he's better at giving advice than my parents. (Ling, 12 years old)

When British Chinese children can rely on other individuals for support, advice and comfort, the lack of intimacy between the parent and child dyad was not viewed as problematic.

I'm just their son; parents are just there to look after you. I find it easier to talk to my sister than my parents, if there are any problems I would speak to her first. (Adam, 12 years old)

## Changes in intimacy levels

In contrast to parents’ upbringing, nine contemporary British Chinese parents reported a much more direct and expressive style of parenting with their own children now. Parents demonstrated an open display of love and affection through hugging and kissing, as well as praising children more generally. Chinese parents suggested that tangible forms of intimacy could enhance feelings of closeness between the parent and child dyad. As Gabb (2008) suggested, bodily encounters in families can express many different feelings such as love, gratitude, compassion and remorse, and add a physical dimension to intimacy levels between parents and children. The majority of parents did not feel embarrassed to demonstrate their feelings through physical acts unlike the previous generation.

I think Chinese people feel embarrassed to show their emotions, especially the older generation, like my mum and dad, they never did that. You see, Chinese people always like to keep things inside; they never show their feelings. But I want to show my children affection and how I am feeling. I think body language and affection are really important. (Heather, early forties, Chinese mother)

However, British Chinese adolescents suggested that hugging and holding hands with parents was embarrassing or unnecessary as they grew older. Adolescents mentioned that they preferred other forms of intimacy as expression of care such as talking to parents and sharing time together. Similarly, contemporary British Chinese parents raised the importance of spending time together, which differed from their own childhood experiences.

I never saw my parents growing up, especially my dad as he was trying to earn money to improve the family and to provide a better living. When all the family came over to the UK, yeah the family was reunited but my parents still had to work hard, to save more money up and to pay for things. (Ava, mid-forties, Chinese mother)

The issue of global relocation and the relationship between parents and children prior to, and after, migration can impact upon contemporary British Chinese parent–child bonds. Before migrating to Britain, Grace (aged 14) explained that she was mainly cared for by the housekeeper and her grandmother in Hong Kong, as both her parents were busy working. As a result, Grace did not feel close to her parents whilst growing up in Asia. When moving to England with her mother in her early adolescence, the lack of intimacy continued in the mother–child relationship and the distance between them remained.

When we were in Hong Kong, I wasn't really close to our parents anyway because the maid and my grandma used to look after us, so we have just stayed in trend here … So we're [mother and daughter] not very close, we don't understand each other that much. I don't talk to her that much, or know what she thinks. (Grace, 14 years old)

In contrast, Sue (early fifties) suggested that the mother–son relationship had improved since moving to the UK from Hong Kong (since 2002) because of the cultural differences for intimate relating between parents and children.

After coming to the UK, I feel closer to my son because of the differences in culture. Like, the manner is different here and we can be more affectionate, do things like cuddling and talking more intimately. I feel that this will prolong our relationship also. (Sue, early fifties, Chinese mother)

Eight of the British Chinese parents invested heavily in their relationships with children, and promoted friendships, feelings of trust, openness and support within the dyad. Children seemed appreciative of their parents’ efforts to establish a close bond with them.

If you're parents are really close to you, they understand you, and the way that you act, so you don't have to explain things. (Casey, 12 years old)

Fathers in both Western and Chinese societies are viewed as being more likely to impose their authority on children (Yee, 2005), especially with adolescent daughters due to concerns around possible sexual activities around this age (Regnerus, 2006). As a result, adolescents may limit their communication with their fathers, be more defensive and guarded towards them, therefore creating distance within the parent–child relationship (Prager, 1995). However, the Chinese fathers in this sample expressed the desire to be more caring and nurturing, and less emotionally absent and authoritarian, compared to the previous generation. Such views coincide with modern fatherhood discourses in the West (Finn & Henwood, 2009) and family changes within some Eastern contexts also (Chan, 2012).

I like to offer myself as a friend to my son, as he gets older I don't want him to just treat me as a parent who only issues authority and demands things. I want him to be able to trust me and to tell me problems, ask for assistance whenever he has problems with his work or schoolwork. I want him to know I'm there for him as a friend, to play with or to play games with. We are basically there to support each other. So our relationship is growing all the time, hopefully to be a stronger bond. (Ting, early forties, Chinese father)

Furthermore, British Chinese fathers did not shy away from open lines of communication with their offspring regarding various topic matters.

There are many things that I feel comfortable discussing with my daughter. Even things they prefer to discuss with their mother, like adult issues and growing up. (Edmund, mid-fifties)

Chinese fathers’ emotional openness and availability to children contrasts the traditional Chinese doctrine, where fathers should be stern, whose duty it is to raise the family, whereas the mother's role is to be an affectionate caretaker (Parke et al., 2005). The British Chinese fathers’ estimation of their own advisory role and friendly approach corresponded with the children's perspectives of the father–child relationship. Even when physical distance separated some households, some British Chinese children still felt close to their fathers. Grace, for example, felt close to her father even though he worked in Hong Kong.

Even though I don't see him much, he does understand me. He understands me more than my mum. He's more like a friend than a dad really … He doesn't know me in detail, in what I like or stuff cause he used to work loads while I was young as well, and now I see him even less, but he understands me. (Grace, 14 years old)

Children's comments correlate with the existing literature, where intimate relations between parents and children consist of ‘deep knowing’ and understanding of the child (Jamieson, 1999). The experience of being understood and accepted by someone in a positive manner seems to capture what is important and rewarding about intimate experiences and tends to elicit trust between the relationship partners (Prager, 1995). Children's comments contrasted Chinese parents’ experiences whilst growing up.

I feel as though my daughter will be able to confront me with her problems more than I did with my parents, because my parents were not as open … I am more Westernised than my parents were. So I can talk to my children more and they can talk to me and confide in me, so it's a lot easier than the relationship I had with my parents. (Ava, mid-forties, Chinese mother)

Whether it is with the mother or father, adolescents appear to cope much better when they feel accepted by their parents and are able to talk about their problems and issues (Noller & Callan, 1991). However, despite the emphasis placed upon ‘being like friends’, parents still expected children to respect and obey them. Here the Chinese value of filial piety was still seen as important for family functioning and children's socialisation. Unbeknown to parents, children suggested that the existence and continued emphasis of filial piety could create distance within the parent–child relationship, despite parental efforts to encourage intimacy levels.

My parents are serious, you don't have a joke with them, you do as they say – listen and obey. (Adam, 12 years old)

Four British Chinese parents felt caught between traditional Chinese ideals and Westernised notions of equality and intimacy, which supports Yee's (2005) research regarding Hong Kong children and parents. Like the older generation, British Chinese parents found it difficult to be self-disclosing and did not support the idea of equality within the parent–child relationship due to Chinese cultural values (e.g. the importance of inhibiting emotions and adult authority). British Chinese children themselves were fully aware of the deeply ingrained nature of their parents’ adherence to some aspects of Chinese culture.

I don't think she would understand, I think she is the way she is [with traditional Chinese values] for quite long so I am not confident that she is going to change, it's a habit. (Ken, 11 years old)

For British Chinese children who had authoritarian parents, they felt that they had a less intimate relationship with their mothers and fathers as a result.

There's still a massive gap between us [parents and child]. Because I don't share my feelings with them or anything because I just don't want to. I just keep it inside me. (Olivia, 12 years old)

Six British Chinese adolescents (daughters and sons) reported that their mothers were ‘old-fashioned’ and ‘strict’ and less accepting than fathers, who in some cases were viewed as more relaxed and modern. As a result, British Chinese mothers were less likely, or perceived to be less likely, to accept or understand certain issues raised by young people. Mismatches in opinions and viewpoints between British Chinese mothers and adolescents led to misinterpretation, disagreements and conflicts, as well as reduced levels of intimacy between the parent–child dyad.

I don't bother telling my mum about my friends or lads … I think I would be closer to my mum if she understood my personal life more. (Charlotte, 14 years old)

## Adolescence and parent–child closeness

The time between one's childhood and adulthood, otherwise known as adolescence, is commonly conceived as an ever-changing context for parents and young people alike. During this time, responsibilities, rights and privileges for the adolescent may be unclear for both parties (Noller & Callan, 1991). However, pubertal changes have been cited as eliciting changes in the parent–child attachment. Prager (1995) suggested that the increasingly adult-like appearance of post-pubertal adolescents might prompt parents to think of their children as older and more responsible. The parents’ altering perception of the child may then initiate a new level of egalitarianism in the adolescents’ relationships with parents. As Ava (mid-forties) commented:

Although it has only been a few months since we last spoke there have been changes. My daughter has grown up physically and is becoming a young woman. She knows how to look after herself on that front and she knows she is a bigger girl now. So she is becoming more mature and responsible.

However, some parents in the sample suggested that their child still looked ‘young’, were still ‘their babies’ and had ‘not grown up yet’, as such, treated them as immature, much to the young person's disgruntlement.

I guess they think I am the same person as when I was younger. But ever since I was ten I started changing and I act differently … At times I just want to tell my parents ‘don't worry about me so much’ and ‘just leave me alone’. But I don't feel like telling them. They still treat me like a kid that's the main problem. (Olivia, 12 years old)

Adolescents’ desires for greater autonomy and agency are often characterised by their independence from parental authority and greater cooperation and mutuality within the parent–child relationship (Buhl, 2008). In establishing agency and self-reliance, distance may be created within the parent–adolescent relationship (Prager, 1995), where a ‘comfortable zone’ is formed between the parent and child dyad (Nøvik & Solem, 2003). Terms such as ‘breaking away’ or ‘breaking free’ are often used to describe the changes in the young person's relationship with their parents (Arnett, 2010). Nine British Chinese parents raised concerns about the increasing distance placed between children and themselves and the implications this had on their relationship.

When our son starts arguing I find it upsetting but you can't let them have their way. I find it upsetting because to us [as parents], we have a certain standard, but to him, if his standards are different to ours or if he isn't convinced that our standards are better, than it is very difficult for us to relate and that's quite sad. (Louise, late forties, Chinese mother)

Some research has found that adolescents limit their disclosure and confidences to parents in order to protect themselves from unwanted parental supervision and authority (Solomon, Warin, Lewis, & Langford, 2002) and avoid parental reprimands, constraints and conflicts (Prager, 1995). Similar findings can be seen within the British Chinese children's accounts.

There are certain things that you don't tend to mention, or want to mention … Things that I shouldn't have done, misbehaving at school, girls, I mean why would you want to? (Ben, 14 years old)

Solomon et al. (2002) suggested that the withholding of information allows privacy, power and identity for the adolescent, but it is at the expense of parent–child intimacy, as self-disclosure seems to be a central component of intimacy (Prager, 1995).

With the onset of early and middle adolescence, young people who feel a need to gain independence from their parents may attach more value and importance in belonging to their peer group (Adams, 2005). Five British Chinese parents complained that their child's social life, and the increasing importance attached to socialising, was preventing opportunities for family time together.

Of course you want to spend more time together but when they grow older they have their own friends. They won't say ‘oh lets go there’, you have to ask them the night before, if they say yes then it's no problem. If they say ‘I'm meeting someone’, I say we only go out once or twice a week, why can't you meet your friends later? But my husband says if they don't want to go, then leave them. (Heather, early forties, Chinese mother)

Parental resistance of adolescent independence and attempts to maintain total control is likely to result in high levels of conflict, adolescent frustration and a complete breakdown of the parent–adolescent relationship (Noller & Callan, 1991). Accounts from Joy, Olivia and Adam (all aged 12) suggested feelings of being ‘trapped’, ‘not in control’ and ‘not respected’, as a result of parental restrictions, and this created distant parent–child relationships. In support, Smith (1980) argued that the adolescent's perceived lack of parental recognition as a person, and lack of influence within the parent–child relationship, would negatively affect parent–child intimacy levels.

I don't start a conversation with my parents. I just keep my mouth shut when they're in. I don't like talking to them at times. (Olivia, 12 years old)

Adolescents are more likely to engage in verbally intimate interactions with their parents, if mothers and fathers support their efforts to become independent, resulting in better quality child–parent relationship (Noack & Buhl, 2004). Specifically, Spencer (1994) found that parents who listen to adolescent's explanations and attributions without any prejudgements were most likely to elicit frequent and intimate self-disclosures from their children. Feelings of connectedness through conversations allow parents and adolescents to learn more about each other and each other's viewpoints as well as coming to a better understanding when disagreements or conflicts arise. An appreciation of the parent–child relationship by adolescents could promote good feelings towards parents and enhance levels of closeness.

With my friends, sometimes they can't tell their parent anything … But with my parents I can tell them anything. (Carly, 13 years old)

Interestingly, in some families, when disagreements and conflicts occurred, it could help develop levels of intimacy between British Chinese parents and children, as it provided the context for exploring each other's feelings and opinions as well as encouraging mutual understanding. As British Chinese father Ting (early forties) suggested, ‘We [son and father] have the occasional arguments which have helped us to understand each other more’. In such ways, the adolescent does not necessarily leave or break away from their relationship with parents during adolescence (as mentioned earlier), but instead both parents and adolescents can be seen to negotiate and renegotiate new roles in the family through day-to-day exchanges (Robin & Foster, 1989). In other words, parent–child relations are merely transformed during the transition to adulthood and thereafter rather than being totally eliminated.

## Cohesion and family activities

Intimacy within parent–child relationships often requires cohesiveness. Cohesiveness is described as togetherness and the sharing of time and activities within a relationship (Prager, 1995). Cohesive activities, such as sharing a meal, completing a task together (agentic cohesiveness) or watching sports game together (communal cohesiveness), may not necessarily involve intimate engagement, but often serves as a backdrop for intimate interactions. Many parents emphasised the importance of cohesiveness through ‘family time’ and tried to encourage shared activities with children to increase or maintain bonds.

Home life is very important to me. I think family is very much the foundation of society. You learn what's right and wrong and about law–abiding relationships, so it is important. It is important that I believe that we spend enough time together as a family. (Edmund, mid-fifties, Chinese father)

Eating at least one meal together in the day was an example of family time in the contemporary British Chinese household. Despite the differences in family lifestyles and commitments, family meals were repeatedly highlighted and prioritised by British Chinese parents as a way of maintaining contact and closeness within the family.

The main thing is to have dinner together; it's the time to talk about what's happened in the day and to communicate. (Sue, early fifties, Chinese mother)

Such views are in line with Gabb's (2008) study, where sharing food provided time and space for intimacy, whilst helping to create and maintain relationships. In addition to regular meals together, parents and children engaged in shared interests and activities such as sporting activities, hobbies and watching similar television programmes.

I think it's more fun now, because our son can actually take part in some of the sports that we [as husband and wife] do and we don't worry that he's going to get injured. (Ting, early forties, Chinese father)

Family holidays and trips away were also mentioned as valuable ways of spending time together.

Before the house renovations, we used to go on holiday every year like Spain or we would hire a caravan to spend time with the children. My parents didn't do that. We didn't go off anywhere as family leisure time. (Ava, mid-forties)

When similarities in shared interests and activities do occur between parents and children, this can create closer parent–child relationships.

My relationship with my daughter is like friends, because my heart is young, so we think of the same things and do the same. For example, we watch ‘Bring It On’ together; it's a film about dancing. Of course I don't wear the same things as her though! (Sandra, late forties, Chinese mother)

Things like, when I have a boyfriend, I used to not tell her but now I do because you're older and they've been through it all and you can talk about it. (Sophie, 14 years old)

Sanderson (2004) suggested that any attempt to create and maintain intimacy within a relationship is a worthwhile venture, as intimacy levels play an important role in predicting relationship satisfaction and helps to maintain attachments over time. However, parental workload was repeatedly raised by British Chinese parents and children as a barrier to cohesive family activities. This was especially problematic for two parents who worked in the takeaway trade and for two of the lone parent households. Children of such families suggested that they lacked quality time (and thus bonds) with their parents and would like to spend more time with parents.

Sometimes my mum is busy at work, so I don't see her much. I miss communicating with her. Maybe if she spent more time with me I would speak to her more. (Casey, 12 years old)

Children, whose parents worked in other occupations, also felt that parental employment reduces family time together. However, young people understood that paid work was essential for family functioning and well-being and did not complain to their parents as a result.

I know my parents are working very hard, and they are doing it so they can keep us in good schools and stuff like that, so I don't think it is appropriate to say to them that I think they should spend more free time with us. (Ben, 14 years old)

Reduced opportunities to spend time together with parents may not necessarily have a detrimental impact upon the parent–child relationship and levels of closeness however, as quality time is not dependent upon quantity. Instead, quality time is dependent on having opportunities to interact with someone with whom there is a trusting or intimate type of relationship and having opportunities to communicate with one another (Gabb, 2008).

## Discussion and conclusion

Parent–child intimacy was seen to be especially important for the majority of British Chinese parents. Reasons included the lack of affection and intimacy that parents had experienced themselves as children, and because of contemporary parenting outlooks (e.g. being authoritative rather than authoritarian like their own parents were). In terms of encouraging and maintaining intimacy with their children, a majority of British Chinese parents were able to assimilate and accommodate their traditional Chinese values with wider cultural goals and norms. For example, parents were friendly and open with children (prioritised within Western conceptualisations of intimacy) whilst emphasising parental respect and authority (seen within Confucian ethics). Intimacy appears to be encouraged and sustained within the parent–child relationship according to parents’ own cultural preferences, thus depicting a culturally hybrid approach to parent–child intimacy. However, some parents who classified themselves as traditionally Chinese struggled with the conflicting notions of intimacy within individualist and collectivist ideals. Here British Chinese parents appeared to fulfil their traditional responsibilities as authority figures and socialising agents (as deemed appropriate within collectivist values), whilst struggling to display overt displays of love, companionship and mutual enjoyment with children (as seen within individualist or Westernised ideologies). Parents viewed as traditionally Chinese (i.e. strict and authoritarian) were perceived to lack warmth and emotional expressiveness. As such, children (and parents in their own childhoods) felt a lack of intimacy with parents and did not initiate or encourage intimacy with them as a result. Generally, low levels of intimacy correlated with children feeling dissatisfied with the parent–child relationship and resulted in less self-disclosure.

Adolescence was said to affect the intimacy levels between British Chinese parents and children. Indeed, many British Chinese mothers and fathers suggested that their children were ‘growing up’ and this had created distance within the parent–child dyad. Reasons for this included the increasing importance attached to children's peer groups, growing levels of independence from parents and adolescents’ changing personal outlooks. Distance may also be caused by the child's decision to seek support and advice from other networks such as friends and other family members during this time. On the other hand, some contemporary parents suggested that intimacy within the dyad had improved since adolescence due to the child's growing maturity levels and increasing relatedness and communication between the pair. Arguably, then, parent–child relationships were merely transformed during adolescence, and not completely eliminated as some writers would suggest. In support, existing research has shown that parents often remain as close confidents, consultants and advisors to adolescents, and provide general levels of companionship (Prager, 1995). A balance between closeness to parents and a sense of individuality by the young person seems to be important for healthy adolescent development and intimate parent–child relations.

The contrasts and similarities of past and present Chinese family life and an understanding of parent–child closeness have contributed towards a better understanding of the British Chinese household, which also recognises the fluidity of British Chinese families and the agency of children. By listening to both British Chinese children and parents, it demonstrates how cultural explanations can mask the heterogeneity in social actions among members of the same culture (Rothbaum et al., 2000). By viewing parent–child relationships as an interactive and reciprocal process, where intimacy levels can fluctuate and are dependent upon the partners and interactions involved (and not culture alone), we can see how British Chinese parents and children mutually contribute towards the levels of intimacy towards one another in their day-to-day lives.

Despite the findings, it is recognised that there were several limitations to this study. First, the research was on a small scale due to its qualitative nature. Qualitative research itself has been criticised on the grounds of its generality and transparency (Bryman, 2004). Additionally, the researchers’ interpretations of trends in behaviour may be particularly subjective, varying according to philosophical stance, time and location, and the particular subgroups involved (Silverman, 2011). It was therefore essential that the researcher reflected upon factors such as gender, race, ethnicity, class, status and age in order to ‘listen beyond’. Listening beyond is to retain a critical awareness of what is being said and to be ready to explore issues in a greater depth (Mason, 2002). The need to ‘understand beyond’ was also necessary, where researchers should be aware of how social and cultural factors, which both parties bring, can affect the research encounter (Allan & Skinner, 1991). By recognising that researchers are crucial participants in the research process, and through the process of reflexivity as critical thinking, researchers can begin to address their own assumptions and understand how personal feelings and experiences may influence a study. The use of a reflective diary was particularly helpful for such purposes.

Qualitative research has also been criticised in terms of reliability. However, using repeat interviews allowed interviewee's to reflect upon and clarify the researcher's analysis over a period of time. Such a method also permitted families to extend upon their comments in their subsequent interviews (member checking). Regular meetings with university supervisors and various opportunities to present preliminary findings at seminar events permitted peer checking of the results and enabled further reflections by the researcher. Peer checking and member checking is a useful criterion for judging qualitative studies argued Shek et al. (2005). Despite the small-scale nature of the research and the limitations within the study, it is hoped that the findings not only increases knowledge of British Chinese households, but will also contribute towards the necessary future studies to come.
